# High-Performance Optical PET Analysis via Non-Isothermal Crystallization Kinetics

**DOI:** 10.3390/polym14153044

**Published:** 2022-07-27

**Authors:** Dezhi Qu, Jiayang Cai, Fei Huang, Jinyu Zhang, Huajiang Zuo, Shuai Sun, Jinghua Liu, Yongping Bai

**Affiliations:** 1 College of Biological and Chemical Engineering, Guangxi University of Science and Technology, Liuzhou 545006, China; cjy193677464@163.com (J.C.); 18897510476@163.com (F.H.); zhangjinyu2233@163.com (J.Z.); zuohuajiang1984@163.com (H.Z.); 2 Guangxi Key Laboratory of Green Processing of Sugar Resources, College of Biological and Chemical Engineering, Guangxi University of Science and Technology, Liuzhou 545006, China; 3 Wuxi HIT New Material Research Institute Co., Ltd., Wuxi 214000, China; baifengbai@hit.edu.cn; 4 School of Chemistry and Chemical Engineering, Harbin Institute of Technology, Harbin 150001, China

**Keywords:** optical PET, non-thermal crystallization, kinetics

## Abstract

The optical properties of PET have always been a problem that related research has been trying to break through. In the previous work, we modified PET by adding PSLDH (phosphate antioxidant) to obtain a PET film with excellent optical properties. Through non-isothermal crystallization kinetic analysis of modified PET, we hope to verify the conclusion of optical properties by the effect of PSLDH addition on the crystallization properties of PET. PET and PSLDH modified PET were tested by DSC at different cooling rates. The non-isothermal crystallization kinetic process was calculated and analyzed by Jeziorny and Mo methods and the non-isothermal crystallization activation energy was analyzed by Kissinger and Friedman methods by analyzing the DSC curves. The results show that the addition of PSLDH at 0.05 wt% can make the crystallization of PET smaller and slower, which is the same as the case required for excellent optical properties. At the same time, the results can also guide the processing of the optical PET film.

## 1. Introduction

With the rapid development of the manufacturing industry, a variety of polymer materials with excellent properties have emerged. Polyethylene terephthalate (PET) has good prospects in biomedical, textile, electrical, packaging and other fields because of its regular molecular structure, easy crystallization and orientation, good folding resistance, heat resistance, insulation, low price and other advantages [1,2,3]. Therefore, we need to study the crystallization mode and process of polymers, as well as the rate, time, and temperature [4]. The relationship between molecular structure and polymer crystallization dynamics research methods is divided into isothermal crystallization and non-isothermal crystallization. In the actual production process, polymer synthesis is mostly produced and processed by extrusion, injection molding and other methods under non-isothermal and dynamic conditions. Therefore, in order to obtain polymers with expected performance, Many researchers have devoted themselves to studying the non-isothermal crystallization kinetics of polymers, controlling the crystallization rate and crystallinity of polymers through effective synthesis methods to control their morphology and properties [5,6,7,8,9]. Differential scanning calorimetry (DSC) is usually used for non-isothermal crystallization analysis. The methods commonly used to analyze non-isothermal crystallization kinetic parameters of polymers include Ozawa [10], Jeziorny [11,12], and Mo [13,14,15] method. 

In our previous work [16], an optical PET with excellent properties was prepared by in-situ polymerization with the addition of phosphate antioxidant (PSLDH). PSLDH was fabricated by melting blending the P antioxidant (typical phosphite antioxidant, bis(2,4-di-tert-butylphenyl) pentaerythritol diphosphate) with SLDH (LDH (magnesium–aluminum layered double hydroxide) modified by SDS (sodium dodecyl sulfate)) at SLDH/P ratio of 5–40 wt% under nitrogen atmosphere. We synthesized PET resins with different SLDH/P ratios. According to the performance results, when the SLDH/P ratio is 10/90, optimal optical properties can be achieved. At the same condition, the addition amount of PSLDH is 0.05 wt%; however, the paper does not show why the best optical properties can be obtained when the addition amount is 0.05 wt%, and the effect of the cooling process on the optical properties of PET is not considered.

Usually, non-isothermal crystallization kinetics is used to analyze the effect of different additives or polymers on crystallization. In this work, we used DSC to analyze the non-isothermal crystallization kinetics of PET at a different cooling rate, and try to analyze optical performance by the non-isothermal crystallization. In the previous work, we tried to use the Ozawa method to analyze the system, but due to the large temperature difference, we could not find a suitable co-crystallization temperature for analysis. Jeziorny and Mo methods are used to analyze the results of the non-isothermal crystallization process, and Kissinger [17] and Friedman [18,19] methods are used to analyze the activation energy of non-isothermal crystallization. The conclusion can support the addition of 0.05 wt% to obtain the best optical properties, and provide guidance for the processing of this optical PET.

## 2. Materials and Methods

### 2.1. Materials and Synthesis

#### 2.1.1. Materials

Sodium dodecyl sulfate (SDS) and magnesium–aluminum layered double hydroxide (LDH) were supplied by Sigma-Aldrich Corporation (Missouri, NA, USA). Bis(2,4-di-tert-butylphenyl) pentaerythritol diphosphate (P antioxidant) was purchased from Qingdao Jade New Material Technology (Qingdao, China). Ethylene glycol (EG, 99.8%) and terephthalic acid (PTA, 99.9%) were supplied by the Nanjing BASF-YPC Chemical Co., Ltd. (Nanjing, China) Ethylene glycol stibium (EGSb) was purchased from Shanghai Qi Zhi Chemical Co., Ltd. (Shanghai, China) All reagents are used directly without any purification.

#### 2.1.2. Synthesis

Modified LDH (SLDH) was synthesized through the following route. LDH and SDS were introduced into a 500 mL three-neck flask with deionized water and vigorously stirred at 80 °C for 8 h. The suspension was cooled to room temperature and isolated by filtration and washed with distilled water and ethanol three times. The SLDH was vacuum dried at room temperature and ground into powder.

PSLDH was prepared by melt-blending the P antioxidant with the SLDH at a SLDH/P ratio of 10 wt% at a temperature slightly above the melting point (180 °C) under a nitrogen atmosphere.

PET was synthesized by the condensation polymerization process. PTA and EG with the molar ratio of 1:1.2 were added into a 1L polyester synthesis reactor. EGSb was used as a catalyst with the amount of 0.04 wt% based on the total weight of PTA and EG. The esterification reaction was carried out at 235–265 °C under the nitrogen atmosphere. The reaction system was slowly reduced to 30 Pa within 1h and the reaction temperature was increased to 270 °C for polycondensation. The reaction termination was determined by the power of the motor. The modified PET was synthesized by the same route as PET, the PSLDH (dispersed in EG) was added into the reactor before polycondensation with a different ratio [16]. 

### 2.2. Characterization

The non-thermal kinetics was studied by differential scanning calorimetry (NETZSCH, Selb, Germany, 214 polyma). The temperature measurement under the nitrogen atmosphere was as follows: firstly, the sample is heated from room temperature to 350 °C at the heating rate of 20 °C/min, held for 7 min, and then cooled to 20 °C at a different cooling rate (5, 10, 12.5, 15 and 17.5 °C/min). The exothermic curves of heat flow as a function of temperature were recorded and investigated. After cooling, heating each sample to 350 °C again at a heating rate of 10 °C/min, and study the subsequent melting behavior of the sample at different cooling rates.

## 3. Results and Discussion

### 3.1. Effect of Different Cooling Rates on Non-Isothermal Crystallization Behavior

#### 3.1.1. Non-Isothermal Crystallization Behavior

**Figure 1:**
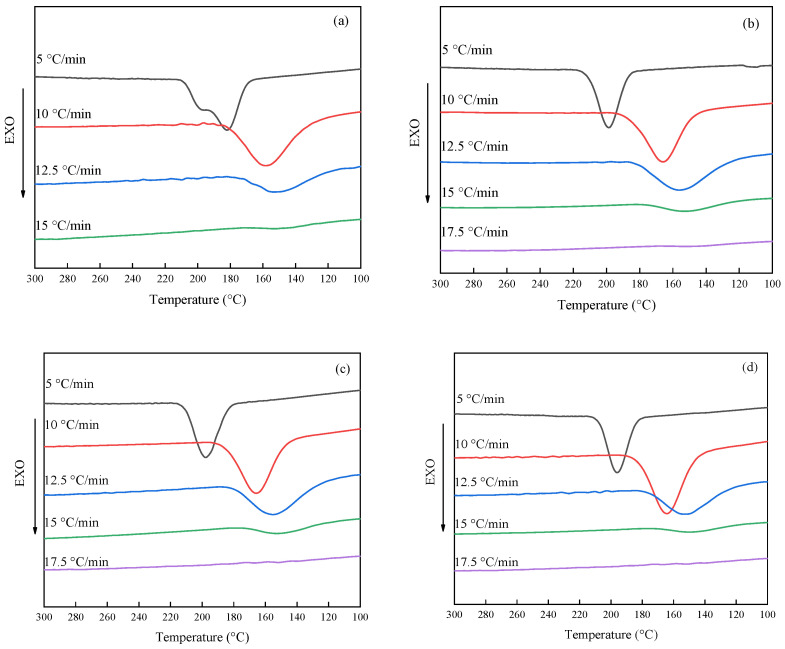

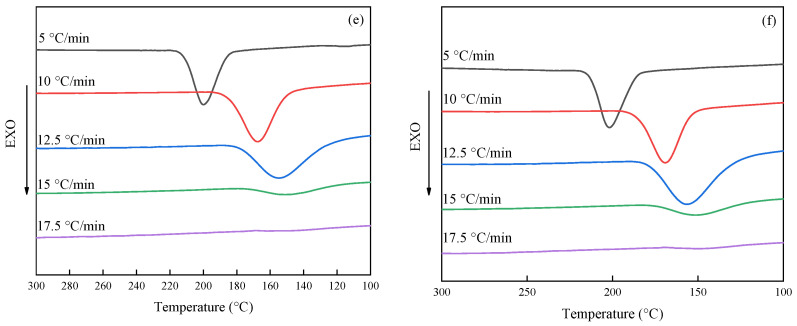
Non-isothermal crystallization curves of PET. (**a**) PET; (**b**) PSLDH 0.01 wt%; (**c**) PSLDH 0.03 wt%; (**d**) PSLDH 0.05 wt%; (**e**) PSLDH 0.07 wt%; (**f**) PSLDH 0.1 wt%.

Non-isothermal crystallization curves of PET and modified PET have shown in Figure 1. The crystallization initial temperature (*T_b_*), the crystallization peak temperature (*T_p_*), the crystallization ending temperature (*T_e_*) and enthalpy of thermal crystallization (Δ*H*) can be obtained by analyzing the DSC curves, and corresponding parameters are listed in Table 1. It can be seen from Figure 1a that an exothermic crystallization peak appears in the cooling curve and the peak splits when the cooling rate is 5 °C/min. PET can slowly crystallize and grow to form large spherulites at a slower cooling rate. These larger spherulites will collide and squeeze with each other at the later stage of crystallization, resulting in the transformation of crystallization behavior. Therefore, the crystallization peak splits. The splitting of the crystallization peak disappears when the cooling rate exceeds 10 °C/min. Indicating that due to the increase of the cooling rate, the spherulite volume of PET decreases, and there is less mutual collision and extrusion in the later stage which causes the peak not to split. The data in Table 1 show that the crystallization initial temperature (*T_b_*) decreases from 209.6 °C to 173.3 °C and the peak temperature decreases from 182.1 °C to 153.6 °C with the acceleration of the cooling rate. In addition, the difference between the crystallization initial temperature and the crystallization peak temperature increases at the same condition. In one word, the increase of the cooling rate increases the width of the crystallization peak; this is mainly determined by the relaxation characteristics of polymer chain motion. During the crystallization process, the molecular chain segment in the melting random coil conformation needs some time to adjust its conformation before it can enter the regular lattice, resulting in a lag period in the cooling crystallization process. The faster the cooling rate, the faster the mobility of the molecular segments decreases, which causes a slower regular arrangement of the lattice and increases the corresponding relaxation time; thus, both the crystal core formation and crystal growth are retarded. Not only the crystallization initial temperature and the crystallization peak temperature are both shifted to the cryogenic region, forming a wider supercooled region, but also the lag phase is relatively prolonged. On the contrary, when the cooling rate is lower, molecular chain motion ability is strong, which accelerated the process of crystal formation and crystallization proceeded in a high-temperature region. The polymer crystallization peak gradually narrowed when the cooling rate continued to increase, and as can be seen from the data in Table 1, the value of crystallization exotherm enthalpy gradually decreased. Indicating that the cooling rate elevation decreased the crystallinity of PET from 42.24 kJ/mol at 5 °C/min to 7.17 kJ/mol at 12.5 °C/min, and even the crystallization peak disappeared at the cooling rate of 15 °C/min. State that the molecular chains of PET are frozen at this time because the cooling rate was too fast to undergo alignment into the crystal lattice. Figure 1b–f are the DSC curves of modified PET with the PSLDH addition of 0.01 wt%–0.1 wt% at different cooling rates, respectively. It can be seen that the crystallization peak of PET narrowed, which is due to the added PSLDH playing a role in the crystal nucleus in the system, leading to the crystallization transformation from homogeneous nucleation to heterogeneous nucleation and promoting the crystallization of PET. Therefore, the crystallization process of PET becomes shorter and the crystallization peak narrow. Meanwhile, PET still shows some crystallinity at a cooling rate of 15 °C/min, demonstrating the promoting effect of heterogeneous nucleation of PSLDH on the crystallization of PET. The peak of the crystalline peak should present an elevated trend with the higher PSLDH addition. But the analysis of the crystalline peak at each addition can see that it presents a tendency of decreasing and then increasing; this is mainly due to the lamellar structure of LDH in the PSLDH having a certain inhibitory effect on the crystallization of PET, which will play hindrance to the crystal growth of PET and slow the crystallization. So that the peak of crystallization is reduced. But its weakening effect on crystallization is not obvious when the addition of more than 0.07 wt%, at which point the improvement of heterogeneous nucleation crystallization is more and more significant due to the addition of PSLDH which is manifested by the enhancement of the crystallization peak. 

#### 3.1.2. Relative Crystallinity (*X*(*T*)) and Temperature (*T*)

Relative crystallinity (*X*(*T*)) refers to the percentage of the crystallized portion at temperature *T* occupying all crystals at the end of the crystal according to the definition of the Avrami equation in the isothermal crystallization process (Equation (1)).

(1) $$X\left(T\right)=\frac{\int_{T_{0}}^{T}\left(\frac{dH_{c}}{dT}\right)dT}{\int_{T_{0}}^{T_{e}}\left(\frac{dH_{c}}{dT}\right)dT}$$

where *T_e_* is crystallization termination temperature, d*H_c_*/d*T* is the enthalpy of crystallization release during an infinitesimal temperature range.

**Figure 2:**
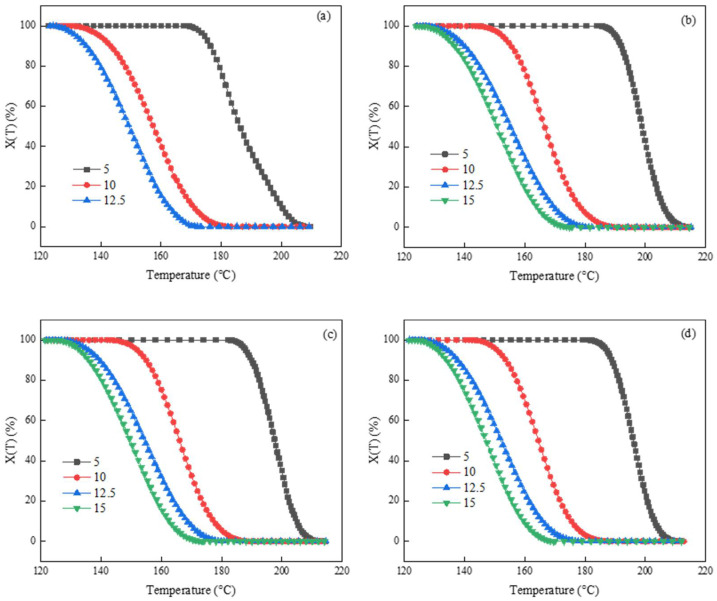

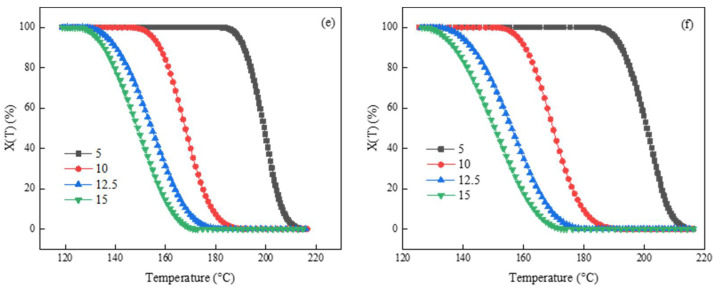
X(T)-T curves of PET. (**a**) PET; (**b**) PSLDH 0.01 wt%; (**c**) PSLDH 0.03 wt%; (**d**) PSLDH 0.05 wt%; (**e**) PSLDH 0.07 wt%; (**f**) PSLDH 0.1 wt%.

By integrating the DSC curve data at a different cooling rate in Figure 1 according to Equation (1), *X*(*T*)-*T* curves (Figure 2) can be obtained. With the increase of cooling rate, the curves move towards low temperature, and the temperature of semi-crystallization and complete crystallization decrease, which is mainly caused by the hysteresis of crystallization and the same as the results of DSC curve in Figure 1. At the cooling rate of 5 °C/min, the crystallization of PET was complete at 170 °C, while the curve at the cooling rate of 12.5 °C/min showed that the crystallization had not started at this temperature; this is consistent with the DSC cooling curve, which reflects the relationship between the mobility of molecular chains and crystallization. Figure 2b–f is roughly consistent with the crystallization of PET and the results obtained above. The addition of PSLDH changes the crystallization from homogeneous nucleation to heterogeneous nucleation.

#### 3.1.3. Relative Crystallinity (*X*(*t*)) and Time (*t*)

*X*(*t*) is the relative crystallinity of the polymer sample at time *t*. Equation (2) can convert crystallization time *t* and temperature *T* according to the cooling rate.

(2) $$t=\frac{\left|T_{0}-T\right|}{\Phi }$$

**Figure 3:**
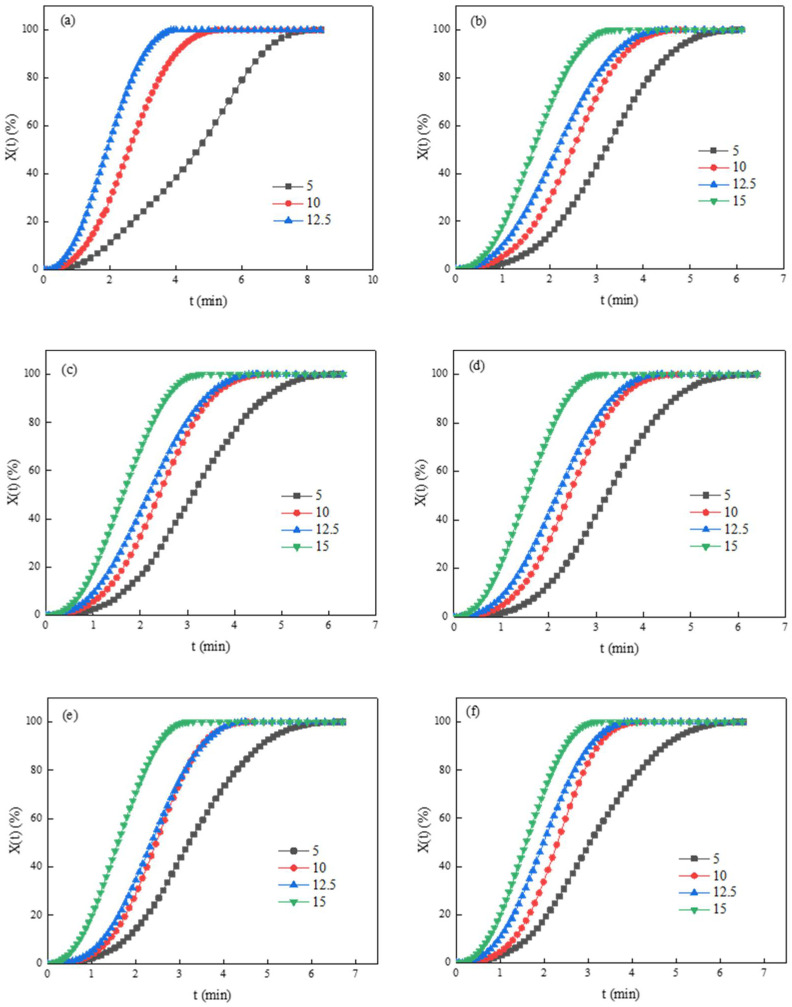
X(t)-t curves of PET. (**a**) PET; (**b**) PSLDH 0.01 wt%; (**c**) PSLDH 0.03 wt%; (**d**) PSLDH 0.05 wt%; (**e**) PSLDH 0.07 wt%; (**f**) PSLDH 0.1 wt%.

Figure 3 is *X*(*t*)-*t* curve converted by Figure 2 *X*(*T*)-*T* curve through Equation (2). It can be seen intuitively from Figure 3 that the crystallization curve is divided into three distinct parts, which correspond to three stages of crystallization with time, nucleation stage, crystal growth stage and crystallization constant stage. At the beginning of cooling down, the system temperature is high, the molecular chain can be quickly discharged into and separated from the crystal lattice, the crystal is not easy to form and the relative crystallinity is low. With the increase of cooling time, when the temperature drops to a certain temperature, the molecular chains are mainly orderly arranged to the crystal lattice, the crystal grows rapidly, and the relative crystallinity increases rapidly in a short time. Further increase the cooling time, the crystallization enters the crystallization constant stage. At this time, as the temperature continues to decrease to the point where the molecular chain segment loses its mobility, the crystal growth stops, and the relative crystallinity remains constant with the increase of time. At this time, the relative crystallinity gradually approaches 100%, due to a large number of crystals in the polyester system, the interaction between spherulites will restrict the moment and folding of the molecular chain which will reduce the folding capacity of the polyester molecular chain. When it is in the constant crystallization stage, the number of molecular chains than can continue to fold into the crystal lattice decreases. Therefore, the relative crystallinity of polyester at this stage increases slowly and the crystal growth stagnates. With the increase of time, the relative crystallinity remains constant. As shown in Figure 3a, the crystal growth of PET resin is very slow until the relative crystallinity increases to about 50%, and then it obviously enters the crystal growth stage, when the cooling rate is 5 °C/min; this is because the homogeneous nucleation of PET is slow in the process of slow cooling, and the crystallization process in the high temperature zone is slow, so it is difficult for the crystal to grow rapidly. It can be seen that the nucleation stage of PET resin has been small when the cooling rate increases to more than 10 °C/min, and the crystallization can be completed in a short time. Compared with Figure 3b–f, the nucleation stage is not obvious when the cooling rate is 5 °C/min. Because the crystallization changes from homogeneous nucleation to heterogeneous nucleation, which can quickly pass through the nucleation agent and enter the crystal growth stage.

It can be seen from the trend of *X*(*t*)-*t* curve that it can clearly reflect the three stage of modified PET crystallization. In the second stage, the relative crystallinity of polyester increases rapidly and the crystallization rate of copolyester is the fastest. With the increase of cooling rate, the slope of the second crystallization stage corresponding to the curve becomes larger and larger; this is because the increase of cooling rate can make polyester form a large number of crystal nuclei in the nucleation stage. The PSLDH can make it easier for polyester to form a large number of crystal nuclei, and make the crystal nucleus generation stage complete quickly. Therefore, in the crystal growth stage, a large number of crystal nuclei will make the relative crystallinity increase faster, and the faster cooling rate can make the temperature reach the easiest temperature for crystal growth faster, so that it can complete crystallization in a short time.

#### 3.1.4. Heating Curves of PET after Different Cooling Rate

**Figure 4:**
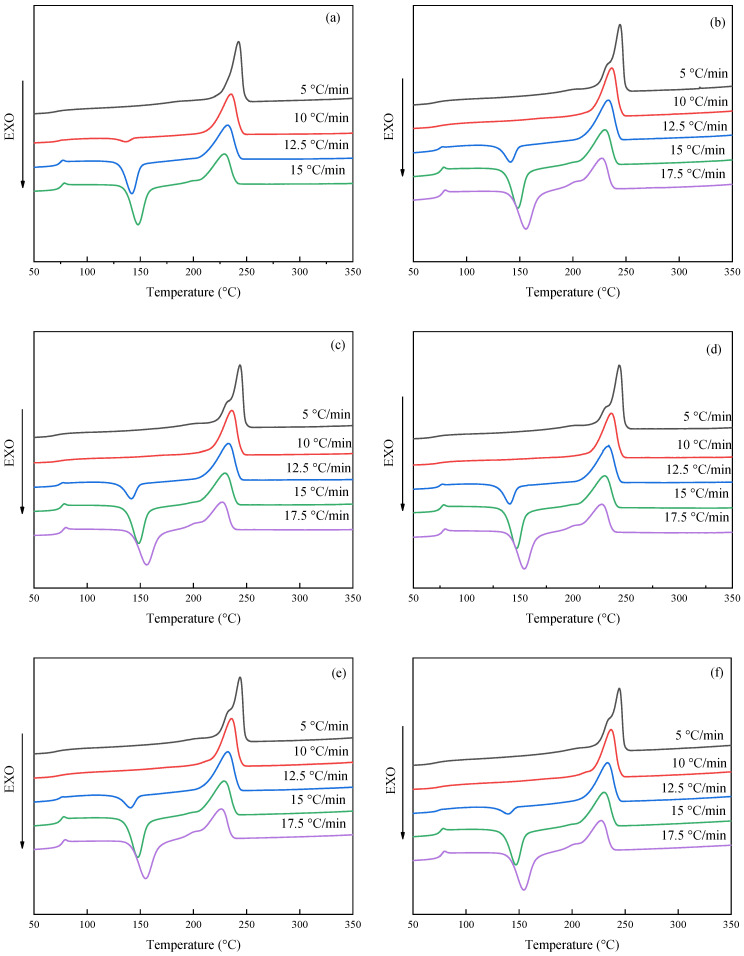
Subsequent heating curves after non-isothermal crystallization. (**a**) PET; (**b**) PSLDH 0.01 wt%; (**c**) PSLDH 0.03 wt%; (**d**) PSLDH 0.05 wt%; (**e**) PSLDH 0.07 wt%; (**f**) PSLDH 0.1 wt%.

Figure 4 Shows the DSC curve at the heating rate of 10 °C/min after cooling at different cooling rates, Figure 4a is PET resin. The curve shows that there is only one melting peak during the heating process when the cooling rate is 5 °C/min; this indicates that PET can be fully crystallized under the cooling rate and there is no cold crystallization peak appears during the heating process, the crystallization melting peak is narrow which indicated the size and molecular chain folding between spherulites are basically same at this time. When the cooling rate exceeds 10 °C/min, the DSC curves show a cold crystallization peak during the heating process, and the faster the cooling rate is, the larger the cold crystallization peak is. Those indicated that the increase in cooling rate makes the crystallization of PET incomplete during the cooling process. When the temperature rises beyond the glass transition temperature, it starts to recrystallize. From the shape of the melting peak, with the increase of the cooling rate, the peak bottom width gradually widens, the initial temperature of the peak decreases, and the peak area of the crystallization melting peak decreases; this is because the crystallization formed at a faster cooling rate does not completely lead to a lower value of enthalpy. At the same time, because there is a cold crystallization behavior in the heating process, the crystallization formed incompletely at the condition which will also lead to the decrease in the enthalpy of the melting peak. The heating curves of modified PET after adding PSLDH change trend are consistent with that of PET resin under the same conditions. But there also have some differences. The heating curve has a shoulder peak at the melting peak when the cooling rate is 5 °C/min, and the value is shown in the corresponding brackets in Table 2; this is mainly because the addition of PSLDH causes PET resin to change from homogeneous nucleation to heterogeneous nucleation, and the integrity of crystallization will be reduced. Therefore, the melting enthalpy of imperfect spherulites produced during crystallization is low when the cooling rate is slow, which is different from that of normal spherulites, so the melting peak during the heating process produces a shoulder peak. Another reason is that the number of crystals produced during heterogeneous nucleation is large after the addition of PSLDH and the large size difference of spherulites due to the slow cooling rate, some smaller crystals can melt at a lower temperature because of their large specific surface area and high surface energy; however, when the cooling rate is increased to 10 °C/min, there is no shoulder peak in the melting peak; this is mainly because there are many imperfect crystals. Although there are differences between imperfect crystals and normal crystals, the distinction between them is not obvious due to the widening of the peak bottom which causes only one melting peak. As PSLDH particles are added to PET, they can be used as crystal nuclei to accelerate the crystallization of PET resin. Therefore, the crystallization can still be completed during the cooling process when the cooling rate is 10 °C/min, and no crystallization peak can be seen in the heating curve.

### 3.2. Kinetics of Non-Isothermal Crystallization

#### 3.2.1. Jeziorny Method

The isothermal crystallization of polymers also can be described by the Avrami equation (Equation (3)) [12].

(3) $$1-X\left(t\right)=\exp\left(-Z_{t}t^{n}\right)$$

*Z_t_* is the isothermal crystallization rate constant, *n* is the Avrami constant which is related to the nucleation mechanism and the crystal growth dimension. Equation (4) is obtained by some transformations from Equation (3).

(4) $$\mathrm{lg}[-\ln\left(1-X\left(t\right)\right]=\mathrm{lg}Z_{t}+n\mathrm{lg}t$$

Thus, the parameters *Z_t_* can be obtained by the intercept and the *n* can be determined *t* of the linear plot of lg[−ln(1 − *X*(*t*))] versus lg*t*. The classical Avrami equation is generally used to describe the primary stage of the isothermal crystallization kinetics of polymers; however, this model is not suitable to depict the non-isothermal crystallization kinetics of polymers.

Jeziorny believes [11] that the initial stage of non-isothermal crystallization can be simplified by Avrami equation at a constant cooling rate. But the non-isothermal crystallization process is more complex than the isothermal crystallization process, and the Avrami equation needs to be modified by replacing *Z_t_* with a non-isothermal crystallization *Z_c_* as follows Equation (5).

(5) $$\mathrm{lg}Z_{c}=\frac{\mathrm{lg}Z_{t}}{\Phi }\;$$

**Figure 5:**
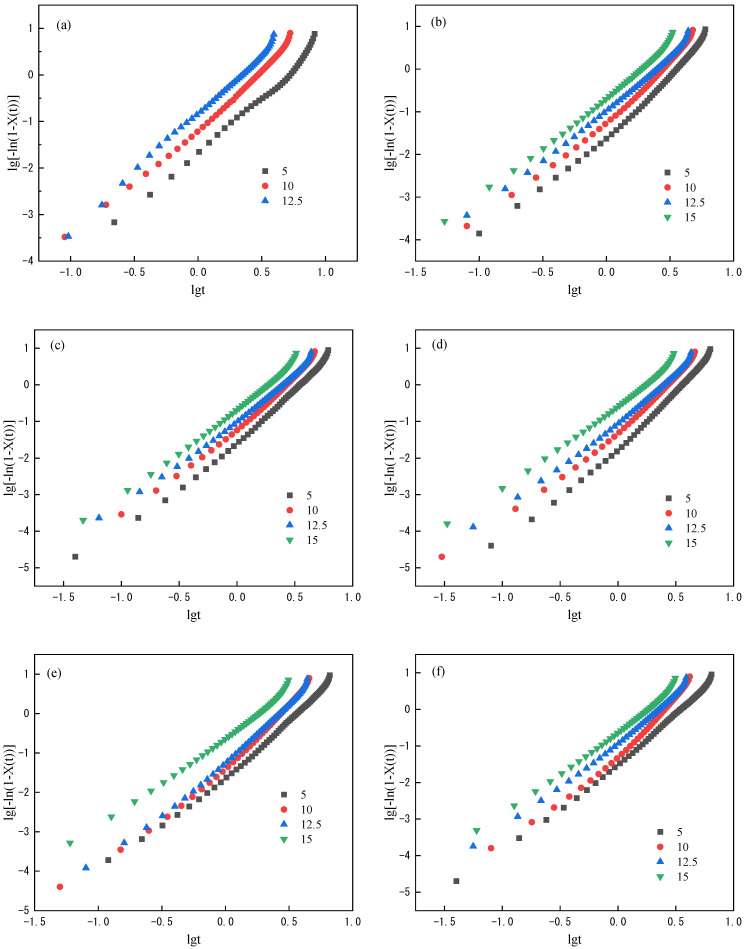
Relationship between lg[−ln(1 − *X*(*t*))] and lg*t* at different crystallization cooling rates. (**a**) PET; (**b**) PSLDH 0.01 wt%; (**c**) PSLDH 0.03 wt%; (**d**) PSLDH 0.05 wt%; (**e**) PSLDH 0.07 wt%; (**f**) PSLDH 0.1 wt%.

Linear fit with lg[−ln(1 − *X*(*t*))] and lg*t* has shown in Figure 5 the Avrami constant n and fitting results are shown in Table 3. The date obtained by from Jeziorny equation basically presents a straight line which indicates the Jeziorny method’s adaptability to the non-isothermal crystallinity process of PET; however, the curve turns slightly when the crystallization exceeds 90%. We analyze the curve separately at the crystallinity of 90%, *n*_1_, *Z_c_*_1_ obtained by fitting the curve with relative crystallinity below 90% and *n*_2_, *Z_c_*_2_ obtained by fitting the curve with relative crystallinity above 90% are listed in Table 3. From the results in Table 3, the non-isothermal crystallization process data of PET has a good linear correlation and high coefficient through the Jeziorny method. The Avrami constant n1 calculated by Jeziorny method fluctuates between 2.277 and 2.283, which indicates the crystal is mainly two-dimensional growth and there are three-dimensional growth and heterogeneous nucleation at the same time. Under the same conditions, the cooling rate constant *Z_c_* increases with the increase of cooling rate, which indicates the increase of cooling rate is conducive to accelerating the crystallization of PET. The Avrami constant value of PET after adding PSLDH shows that the introduction of PSLDH does not affect the growth mode of PET crystallization, which is still two-dimensional growth and there are three-dimensional growth and heterogeneous nucleation at the same time. The cooling rate constant *Z_c_* of PET after adding PSLDH increases with the increase of cooling rate, indicating that the increase of cooling rate also accelerates the crystallization of PET. But the rapid crystallization will lead to the decrease in crystallization perfection of PET, which is consistent with the obvious crystal rearrangement peak of PET after re-heating in Figure 4. 

The curve fitting results of relative crystallinity above 90% show that the Avrami constant n_2_ fluctuates in a wide range from 4.261 to 5.535, indicating that secondary crystallization occurs in the later stage of crystallization which is caused by the collision of growing spherulites in the secondary crystallization stage. The Avrami constant n_2_ of PET resin at a slow cooling rate is larger than that at a rapid cooling rate, which indicates that the spherulites growth mechanism at the later stage of crystallization is more complex, mainly to fill the gap between spherulites; however, the Avrami constant n_2_ of PET added with PSLDH at slow cooling rate is smaller than that at rapid cooling, indicating that the situation at this time is different from the PET resin. Due to the addition of PSLDH, the crystalline size of PET becomes smaller. At the later stage of crystallization, a large number of small spherulites will reduce the voids in the system. Therefore, at the later stage, it is mainly extrusion between spherulites.

#### 3.2.2. Mo Method

Mo has combined the Avrami equation with the Ozawa equation [13]. Equation (2) is shown the relation between the temperature T and the time t under the non-isothermal crystallization. The relative crystallinity *X*(*t*) can be equal to *X*(*T*) under certain conditions which obtained Equations (6) and (7).

(6) lg[−ln(1−X(t))]=lg[−ln(1−X(T))]

(7) lgZ+nlgt=lgK(T)−mlgΦ 

Equation (8) is obtained by organizing Equation (7)

(8) $$\mathrm{lg}\Phi =\mathrm{lg}{\left[\frac{K\left(T\right)}{Z}\right]}^{\frac{1}{m}}-\frac{n}{m}\mathrm{lg}t$$

Let $F\left(T\right)={\left[\frac{K\left(T\right)}{Z}\right]}^{\frac{1}{m}}\;,\alpha =\frac{n}{m}$, we can get Equation (9).

**Figure 6:**
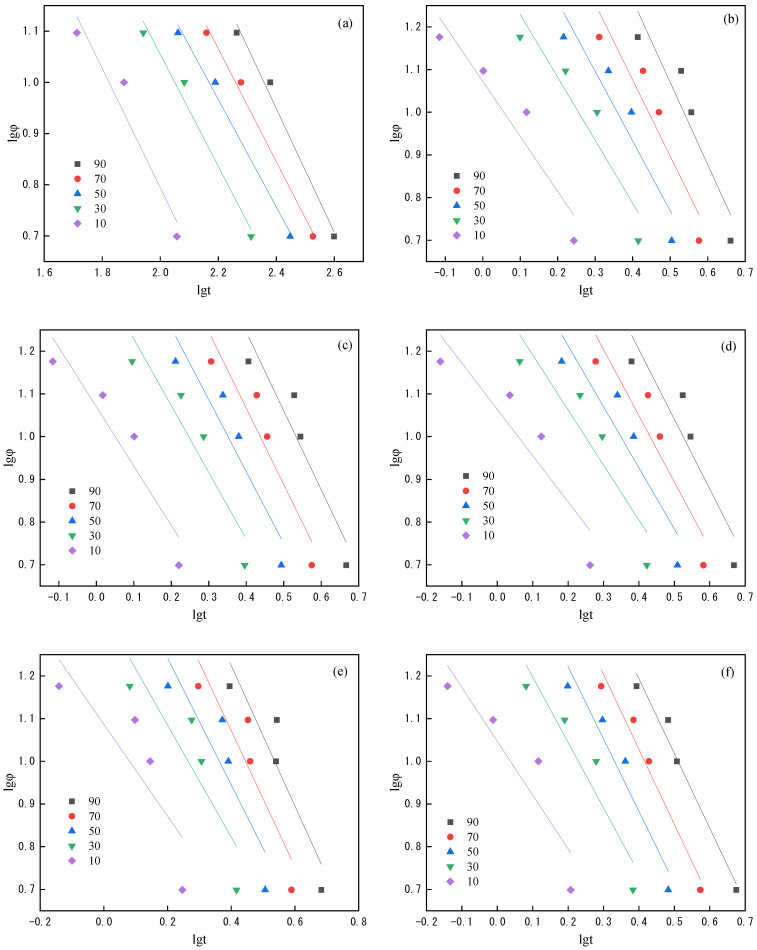
Relationship between lg*Φ* and lg*t* at relative crystallinity of 10%, 30%, 50%, 70% and 90%. (**a**) PET; (**b**) PSLDH 0.01 wt%; (**c**) PSLDH 0.03 wt%; (**d**) PSLDH 0.05 wt%; (**e**) PSLDH 0.07 wt%; (**f**) PSLDH 0.1 wt%.

(9) lgΦ=lgF(T)−αlgt 

where *K*(*T*) is the cooling function, and *F*(*T*) is the value of a cooling rate at which the system is needed to reach a certain crystallinity at a unit time [14,15]. According to Equation (9), Figure 6 is obtained by plotting lg*Φ* vs. lg*t* at relative crystallinity. The data in Figure 6 is linear fitted, *α* and *F*(*T*) can be obtained through the slope and intercept of the straight line (Table 4). It can be seen that the *F(T)* values of PET and modified PET increase with the increase in relative crystallization, which indicates that a higher cooling rate is required to quickly reach a higher relative crystallinity when they crystallize from the melt. Through comparison can be seen that the *F(T)* after the addition of PSLDH is significantly reduced, indicating that PSLDH has a promoting effect on crystallization which is the same as the analysis results above. Comparing the modified PET with different addition of PSLDH can be seen that the *F(T)* is the highest when the addition amount is 0.05 wt%, indicating that LDH has the best blocking effect on crystallization at this addition amount. 

#### 3.2.3. Kissinger Method

**Figure 7:**
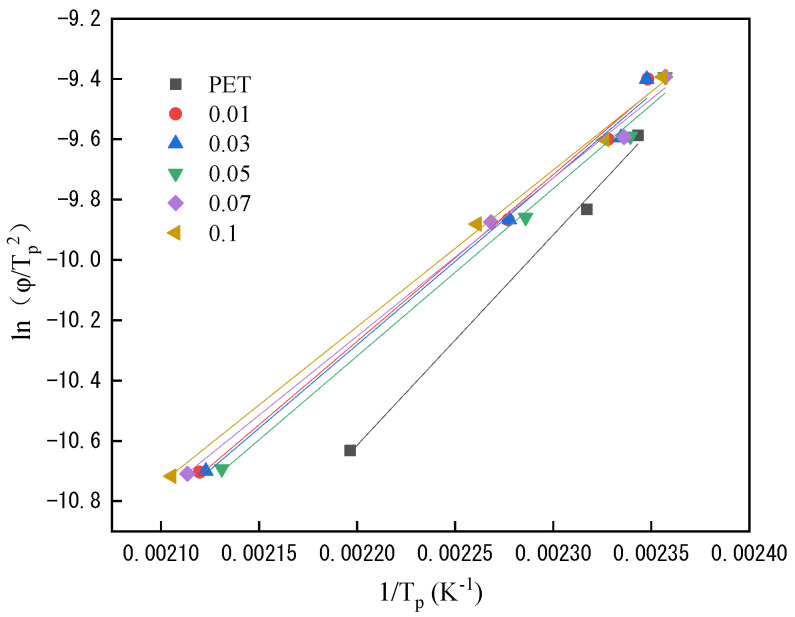
Kissinger plots of ln(*Φ*/*T_p_*^2^) versus *Tp*^−1^.

The non-isothermal crystallization activation energy of the polymer is estimated by the Kissinger Equation (10) [17].

(10) $$\frac{d\left[\ln\left(\frac{\Phi }{T_{p}^{2}}\right)\right]}{d\left(\frac{1}{T_{p}}\right)}=-\frac{\Delta E}{R}$$

where *R* is the gas constant and Δ*E* is the non-isothermal crystallization activation energy of PET. Substitute the *T_p_* corresponding to the cooling rate *Φ* of the aforementioned polyester into the above formula, and plot ln(*Φ*/*T_p_*^2^) against *T_p_*^−1^ to obtain Figure 7 and Table 5. From Figure 7 and data, it can be seen that the crystallization activation energy of PET shows a gradually increasing trend. Since the activation energy calculated by the Kissinger method is to characterize the difficulty of molecular chains entering the lattice, the greater the activation energy, the more difficult for molecular chains to enter the lattice; this indicates that the layered structure of LDH in PSLDH reduces the crystallinity of PET resin, which is consistent with the previous results. According to the pervious results, the increase in cooling rate will make the crystallization peak move towards high temperature. At the same time, the difference between the peaks of different cooling rates will gradually change after the addition of PSLDH. Taking the difference between 5 and 10 as an example, PET is 23.7. After the addition of PSLDH, it shows a trend of increasing and then decreasing with a maximum of 55.9 when the addition of PSLDH is 0.05 wt%; this phenomenon shows that the addition of PSLDH can accelerate the nucleation, but it has a certain blocking effect on the growth of molecular chain; however, the Kissinger method is mainly calculated by cooling rate and crystallization peak value which has limitations, and it is difficult to show the effect of heterogeneous nucleation on the crystallinity of PET from the changing trend of the data. 

#### 3.2.4. Friedman Method

Since Kissinger method is not very accurate in some cases, and Friedman method is considered to be a method for obtaining reliable non-isothermal melt crystallization activation energy values now. The formula [18,19] is

(11) $$\ln{\left(\frac{dX}{dt}\right)}_{X,\Phi }=\ln\left(Af\left(X\right)\right)-\frac{\Delta E}{RT_{X,\Phi }}$$

**Figure 8:**
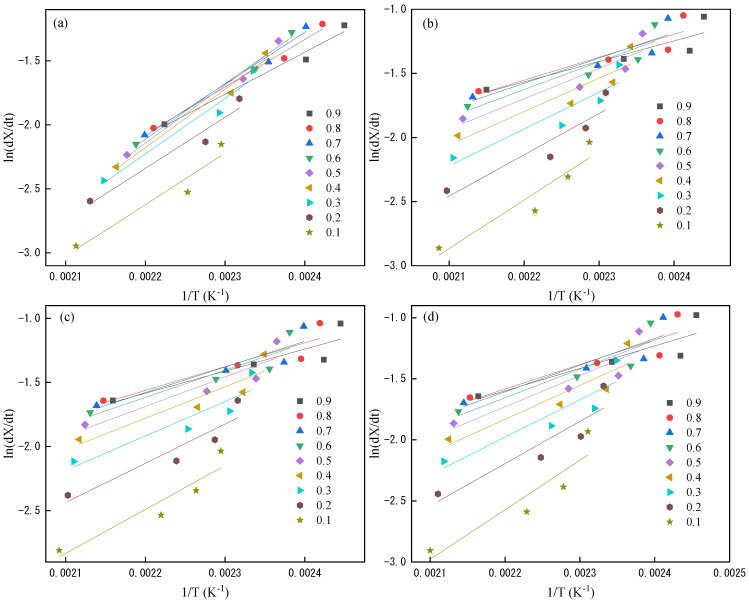

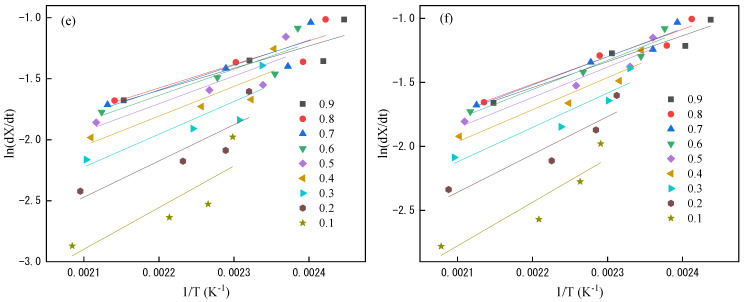
The fitting curve of PET by Friedman method. (**a**) PET; (**b**) PSLDH 0.01 wt%; (**c**) PSLDH 0.03 wt%; (**d**) PSLDH 0.05 wt%; (**e**) PSLDH 0.07 wt%; (**f**) PSLDH 0.1 wt%.

This means that by plotting ln(d*α*/d*t*) versus 1/*T* at certain relative crystallinity, the Δ*E* at this relative crystallinity can be obtained by fitting the slope of the curve in Figure 8. In this paper, the relative crystallinity of 0.1–0.9 is selected for calculation, and the obtained results are listed in Table 6.

It can be seen that the activation energy of PET decreases at first and then increases. The higher activation energy in the early stage is mainly because the crystallization is at the nucleation stage. When the nucleation is gradually formed and stabilized, the activation energy gradually decreases and enters the rapid growth stage of crystallization. The spherulites squeeze each other when the crystallization is basically complete, resulting in a decrease in crystallinity and thus an increase in activation energy. The crystallization activation energy of the PET resin has improved after adding PSLDH which indicates the addition of LDH in the PSLDH has limited the crystallinity of the PET, and it combines the promotion of crystallization by heterogeneous nucleation and the limitation of crystallization by LDH form the changing trend. It can be seen that the initial crystallization activation energy of the PET increases from −33.70 kJ/mol to −15 kJ/mol when PSLDH addition for 0.05%; this is because a large amount of PSLDH is added as a crystal nucleus to promote the crystallization in the early stage of crystallization, but the crystallization activation energy of PET increases rapidly due to the presence of LDH. The crystallization activation energies or several modified resins are basically consistent when the relative crystallinity is 0.9, which means that the crystallization of PET has no time to grow up, and it is basically dominated by small crystals which meet the requirements of optical films for crystallization. In summary, it can be verified that adding 0.05% of PSLDH can obtain the best optical performance, which is consistent with the conclusions of the previously published papers.

## 4. Conclusions

In this work, we analyzed the non-isothermal crystallization kinetics of the previously synthesized modified PET. Through the results of non-isothermal crystallization kinetics analyzed, the addition of PSLDH will promote the crystallization of PET and make the crystal volume smaller. The analysis shows that the increase of PSLDH will improve the crystallization activation of polyester in the later stage which inhibit the crystallization of PET and the Friedman method is better for this system than the Kissinger method. Therefore, a small amount of small volume crystals will be produced when the addition of PSLDH is 0.05%, which can improve the optical properties of polyester. The results are the same as the previous work which provides theoretical support.

## Figures and Tables

**Table 1:** Non-thermal crystallization parameters of PET and modified PET at different cooling rates.

Sample	*Φ*/°C·min^−1^	*T_b_* */°C	*T_p_* */°C	*T_e_* */°C	Δ*H* */kJ·mol^−1^
PET	5	209.6	182.1	169.5	42.24
	10	183.2	158.4	129.3	27.25
	12.5	173.3	153.6	123.1	7.17
0.01 wt%	5	214.9	198.6	184.4	45.40
	10	191.5	166.0	142.7	39.46
	12.5	182.2	156.4	126.2	19.04
	15	175.6	152.7	124.8	4.19
0.03 wt%	5	213.2	197.9	182.0	43.94
	10	190.0	165.9	142.0	36.86
	12.5	181.6	155.2	125.8	17.44
	15	174.1	152.8	124.4	3.517
0.05 wt%	5	212.2	196.1	180.3	44.32
	10	188.8	164.3	141.5	35.97
	12.5	179.4	154.3	124.7	15.53
	15	170.0	151.1	123.5	2.30
0.07 wt%	5	215.4	200.0	181.8	46.61
	10	192.5	167.7	146.0	40.34
	12.5	183.9	154.9	127.9	20.98
	15	172.8	151.1	124.9	3.527
0.1 wt%	5	216.1	201.8	183.4	47.17
	10	192.7	169.1	149.9	40.38
	12.5	180.8	156.7	131.1	21.69
	15	174.2	151.3	126.3	4.54

* *T_b_* the crystallization initial temperature; * *T_p_* the crystallization peak temperature; * *T_e_* the crystallization ending temperature; * Δ*H* the enthalpy of thermal crystallization.

**Table 2:** Value of heating curves after non-isothermal crystallization.

Sample	*Φ*/°C·min^−1^	*T_pcc_* */°C	Δ*H_cc_* */kJ·mol^−1^	*T_mp_* */°C	Δ*H_m_* */kJ·mol^−1^
PET	5	-	-	242.3	43.84
	10	136.5	2.14	235.8	36.82
	12.5	142.2	17.03	231.8	33.76
	15	147.8	24.24	229.0	31.48
0.01 wt%	5	-	-	244.5 (232.2)	46.32
	10	-	-	236.2	40.59
	12.5	141.3	9.55	233.3	38.80
	15	148.2	23.87	230.4	36.12
	17.5	155.7	27.59	227.2	34.40
0.03 wt%	5	-	-	243.7 (232.2)	45.36
	10	-	-	236.1	38.29
	12.5	141.7	8.80	232.3	35.66
	15	148.4	22.92	229.5	33.32
	17.5	156.1	25.82	227.5	30.63
0.05 wt%	5	-	-	243.6 (231.7)	45.61
	10	-	-	235.9	38.48
	12.5	140.8	9.46	233.8	35.72
	15	147.2	22.62	230.0	34.16
	17.5	154.5	25.71	227.4	31.13
0.07 wt%	5	-	-	243.9 (232.6)	48.27
	10	-	-	235.9	41.07
	12.5	141.2	6.79	232.5	38.36
	15	147.5	21.51	228.3	35.33
	17.5	154.9	24.80	226.2	32.92
0.1 wt%	5	-	-	244.4 (234.0)	48.35
	10	-	-	236.8	41.38
	12.5	139.2	3.96	233.3	38.73
	15	147.2	18.64	230.0	35.67
	17.5	154.5	24.33	227.4	33.17

* *T_pcc_* the peak temperature of cold crystallization; * Δ*H_cc_* the enthalpy of cold crystallization; * *T_mp_* the peak temperature of melting; * Δ*H_m_* the enthalpy of melting.

**Table 3:** Non-isothermal crystallization kinetics by Jeziorny method.

Sample	*Φ*/°C·min^−1^	*n*_1_	*Z_c_*_1_	*R*	*n*_2_	*Z_c_*_2_	*R*
PET	5	2.338	0.021	0.999	5.535	0.000	0.993
	10	2.396	0.071	0.998	4.370	0.005	0.981
	12.5	2.479	0.143	0.999	4.784	0.006	0.983
0.01 wt%	5	2.649	0.030	0.996	4.621	0.002	0.987
	10	2.532	0.067	0.995	4.413	0.007	0.987
	12.5	2.386	0.111	0.999	4.596	0.007	0.983
	15	2.363	0.215	0.999	4.909	0.018	0.990
0.03 wt%	5	2.633	0.032	0.996	4.808	0.001	0.988
	10	2.609	0.070	0.997	4.302	0.009	0.989
	12.5	2.401	0.107	0.999	4.614	0.007	0.983
	15	2.385	0.217	0.999	4.782	0.023	0.987
0.05 wt%	5	2.873	0.022	0.996	4.511	0.002	0.987
	10	2.575	0.064	0.994	4.419	0.008	0.989
	12.5	2.475	0.099	0.998	4.780	0.006	0.985
	15	2.277	0.270	0.999	4.945	0.027	0.984
0.07 wt%	5	2.701	0.028	0.996	4.261	0.003	0.986
	10	2.754	0.053	0.994	4.610	0.007	0.989
	12.5	2.747	0.063	0.997	5.117	0.003	0.982
	15	2.291	0.244	0.999	5.075	0.020	0.983
0.1 wt%	5	2.567	0.037	0.998	4.334	0.002	0.984
	10	2.736	0.066	0.993	4.733	0.008	0.992
	12.5	2.439	0.129	0.998	4.738	0.010	0.985
	15	2.310	0.245	0.999	5.028	0.021	0.985

**Table 4:** Non-isothermal crystallization kinetics by the Mo method.

Sample	*X(T)*/%	*F(T)*/K·min^α−1^	*α*	*R*
PET	10	3.123	1.164	0.968
	30	3.242	1.094	0.988
	50	3.275	1.049	0.995
	70	3.492	1.103	0.996
	90	3.852	1.210	0.993
0.01 wt%	10	1.072	1.292	0.951
	30	1.377	1.476	0.942
	50	1.586	1.634	0.942
	70	1.790	1.786	0.942
	90	2.034	1.927	0.938
0.03 wt%	10	1.070	1.070	0.941
	30	1.385	1.385	0.936
	50	1.591	1.591	0.940
	70	1.782	1.782	0.945
	90	1.983	1.983	0.943
0.05 wt%	10	1.064	1.082	0.918
	30	1.320	1.289	0.922
	50	1.499	1.430	0.926
	70	1.669	1.550	0.929
	90	1.857	1.632	0.926
0.07 wt%	10	1.087	1.076	0.846
	30	1.348	1.315	0.879
	50	1.540	1.487	0.896
	70	1.710	1.596	0.915
	90	1.883	1.647	0.926
0.10 wt%	10	1.047	1.262	0.917
	30	1.351	1.535	0.945
	50	1.561	1.696	0.967
	70	1.731	1.757	0.983
	90	1.894	1.750	0.987

**Table 5:** The non-isothermal crystallization activation energy of PET by the Kissinger method.

Sample	Slope	Ea/kJ·mol^−1^	*R*
PET	6973.80	−57.98	0.998
0.01 wt%	5530.80	−45.98	0.998
0.03 wt%	5534.60	−46.01	0.996
0.05 wt%	5553.77	−46.17	0.997
0.07 wt%	5244.52	−43.60	0.998
0.1 wt%	5195.02	−43.19	0.999

**Table 6:** Non-isothermal crystallization activation energy of PET by Friedman method.

Relative Crystallinity (*X*)/%	Δ*E*/kJ·mol^−1^					
	PET	0.01	0.03	0.05	0.07	0.1
10	−33.41	−31.44	−28.55	−33.70	−28.38	−28.28
20	−33.19	−26.81	−25.39	−28.68	−24.69	−24.56
30	−36.02	−23.92	−22.30	−25.47	−22.32	−22.32
40	−37.88	−21.69	−20.40	−23.14	−20.22	−20.83
50	−37.66	−19.70	−19.14	−21.27	−18.86	−19.69
60	−36.09	−17.99	−17.60	−19.62	−17.92	−18.96
70	−33.68	−16.41	−16.66	−18.15	−16.95	−18.29
80	−30.76	−14.90	−15.45	−16.75	−16.08	−17.76
90	−27.20	−13.32	−14.33	−15.31	−15.18	−16.84
